# Uncovering Specific Navigation Patterns by Assessing User Engagement of People With Dementia and Family Caregivers With an Advance Care Planning Website: Quantitative Analysis of Web Log Data

**DOI:** 10.2196/60652

**Published:** 2025-02-11

**Authors:** Charlèss Dupont, Tinne Smets, Courtney Potts, Fanny Monnet, Lara Pivodic, Aline De Vleminck, Chantal Van Audenhove, Maurice Mulvenna, Lieve Van den Block

**Affiliations:** 1 End-of-life Care Research Group, Vrije Universiteit Brussel (VUB), Laarbeeklaan 103, Brussels, 1090, Belgium, 32 484433257; 2 Department of Family Medicine and Chronic Care, Vrije Universiteit Brussel (VUB), Brussels, Belgium; 3 School of Psychology, Ulster University, Coleraine, United Kingdom; 4 LUCAS Center for Care Research and Consultancy, KU Leuven, Leuven, Belgium; 5 School of Computing, Ulster University, Belfast, United Kingdom

**Keywords:** dementia, advance care planning, user engagement, web-based tool, care, website, caregiver, communication, tool, online

## Abstract

**Background:**

Web-based tools have gained popularity to inform and empower individuals in advance care planning. We have developed an interactive website tailored to the unique needs of people with dementia and their families to support advance care planning. This website aims to break away from the rigid pathways shown in other tools that support advance care planning, in which advance care planning is shown as a linear process from information to reflection, communication, and documentation.

**Objective:**

This study aimed to assess the website’s usage by people with dementia and their family caregivers, identify distinct user engagement patterns, and visualize how users navigated the website.

**Methods:**

We analyzed the website’s log data obtained from an 8-week evaluation study of the site. Interactions with the website were collected in log data files and included visited web pages or clicked-on hyperlinks. Distinct user engagement patterns were identified using K-means clustering process mining, a technique that extracts insights from log data to model and visualize workflows, was applied to visualize user pathways through the website.

**Results:**

A total of 52 participants, 21 individuals with dementia and their family caregivers as dyads and 10 family caregivers were included in the study. Throughout the 8-week study, users spent an average of 35.3 (SD 82.9) minutes over 5.5 (SD 3.4) unique days on the website. Family caregivers mostly used the website (alone or with a person with dementia) throughout the 8-week study. Only 3 people with dementia used it on their own. In total, 3 distinct engagement patterns emerged: low, moderate, and high. Low-engagement participants spent less time on the website during the 8 weeks, following a linear path from information to communication to documentation. Moderate- and high-engagement users showed more dynamic patterns, frequently navigating between information pages and communication tools to facilitate exploration of aspects related to advance care planning.

**Conclusions:**

The diverse engagement patterns underscore the need for personalized support in advance care planning and challenge the conventional linear advance care planning representations found in other web-based tools.

## Introduction

Advance care planning is a communication process between patients, families, and health care professionals to “define goals and preferences for future care and treatment” [1]. This process holds particular relevance in dementia [2]. The cognitive decline in dementia highlights the need for early initiation of advance care planning, enabling people with dementia to reflect on and express their preferences for future care and treatments [3-5]. While existing definitions of advance care planning often focus on medical care decisions [6], people with dementia and their families have emphasized that it should include exploring what matters now and in the future, including nonmedical aspects of care [7-10]. Furthermore, people with dementia and their families have expressed a need to discuss future care together [7]. This aligns with the recently introduced public health to advance care planning, emphasizing a shift toward a social focus on “what matters most to people” rather than the current emphasis on end-of-life decision making and underlining the need to support conversations in the family context [11].

To support advance care planning within the family context, interactive web-based tools like websites or apps have been promoted [12]; however, despite their proven benefits in other populations, there is a noticeable absence of tools tailored to the specific needs of people with dementia and their families [13-15]. To address this gap, we developed a website for and with people with dementia and their families [16-19]. This website deviates from the structured linear pathways found in other tools to support advance care planning [15]. Such tools adhere to a stepwise procedure, often commencing with information provision, prompting reflection, followed by communication, and concluding with documentation, commonly in the form of advance directives. For example, in some cases, users must complete or make an explicit effort to skip a step before being able to proceed to the next step, eg, users cannot access certain features such as communication tools or documentation forms without first completing previous tasks, for example, users have to follow a linear pathway when navigating the website [15,20]. In contrast, our website is designed to offer flexibility, allowing users to engage with the content in any way they choose. There are no predefined steps or sequences to follow, and users can access all features, such as communication tools and documentation forms, without needing to complete any previous tasks [16].

Following the development of the website to support advance care planning, we performed an 8-week evaluation study involving people with mild to moderate dementia and their family caregivers [17,18]. The primary focus of this evaluation was to assess the usability, acceptability, feasibility, experiences, and outcomes of using the website [17,18]. Participants in the evaluation study found that the website supports advance care planning [17,18]. After 8 weeks, participants exhibited improved advance care planning knowledge, self-efficacy, and skills [17,18].

Beyond this evaluation, gaining insights into the website’s user engagement and usage patterns is crucial to enhance our understanding of how people with dementia and their families use a website to support advance care planning in the family context, how they engage with it and how single users and dyadic users differ (ie, people with dementia together with their family caregiver). This knowledge will inform enhancements to the website’s design and functionality by highlighting user needs in digital health interventions, ultimately improving support for families navigating advance care planning. Therefore, this study aims to: explore how people with dementia and their family caregivers used the advance care planning support website during an 8-week evaluation study; explore whether and which user behavior clusters can be typified based on the engagement of people with dementia and their family caregivers with the website; and explore and visualize user pathways of the identified user behavior clusters, that is, how the different user clusters of people with dementia and their family caregivers navigate through the website.

## Methods

### Study design

This study quantitatively analyzed web log data from a convergent parallel mixed methods evaluation study of a website designed to support people with dementia and their family caregivers in advance care planning. Web log data was collected during an 8-week evaluation study to capture participants’ interactions with the website, for example, time spent on the website and what pages were visited. This log data was used to explore the website’s usage by people with dementia and their family caregivers, which user behavior clusters can be typified and to visualize user pathways. The protocol, including the objective to explore usage patterns of the website, as well as the main results of the evaluation study, have been published elsewhere [17,18]

### The Development of the Advance Care Planning Support Website

The development of the website followed a user-centred, iterative design process, ensuring alignment with the needs of people with dementia and their families. The website was developed to provide information and support for people with dementia and their family caregivers in advance care planning [16]. Furthermore, the website was designed with flexible navigation options, allowing users to explore different sections at their own pace rather than following a linear, step-by-step approach. This was particularly important given the varying levels of readiness among users to engage with sensitive topics such as future care preferences [8,16].

The website includes advance care planning information, information about legal frameworks, communication tips, and documentation sections. Furthermore, it provides accessibility features, such as text-to-speech and text enlargement and 2 interactive communication tools, an “Interactive Card Tool” based on the recently developed paper-based version of the Levenswensen (Life Wishes) cards and a fill-in tool “Thinking Now About Later,” that guides users through a reflective process to help users think and talk about and write down their preferences for the present and future (MultimediaAppendices 1  2). The results of the evaluation study and experiences with the interactive communication tools, have been published elsewhere [16,18,21].

### Participants, Recruitment and Setting

People with mild to moderate dementia, including both early and late onset, along with their family caregivers as dyads or the family caregiver alone, were recruited [17]. To be eligible, participants needed to meet the criteria shown in Textbox 1 [17] .

Textbox 1. **Inclusion criteria for the study.**

Both people with dementia and their family caregivers:

- Express interest in and willingness to test the website to support advance care planning.
- Provide consent for study participation.
- Proficient in speaking and understanding Dutch.
- Own a device (laptop, tablet, mobile phone, etc).

People with dementia:

- Diagnosed with young- or late-onset dementia.

Family caregivers:

- Actively involved in the care (physical, emotional, social, etc) of the person with dementia.

For participants recruited as dyads, at least one had to be capable of navigating the website. For instance, the person with dementia and the family caregiver could not both have visual impairments or other disabilities hindering interaction with the advance care planning website. Participants were recruited through dementia care organizations and memory clinic neurologists. Eligible participants with a confirmed dementia diagnosis were identified by health care professionals. People with dementia who were interested in the study contacted the researcher, received study information, and, if still interested after receiving the study information, were offered an appointment to perform the eligibility screening and provide informed consent. The recruitment process is described in more detail in the study protocol that is published elsewhere [17]. The study was conducted in Flanders, the Dutch-speaking region of Belgium.

### Data Collection Procedures

Between October 2022 and May 2023, 52 people participated in the evaluation study: 21 dyads, that is, 21 people with dementia and 21 family caregivers, and 10 family caregivers alone. Information on sociodemographic data, including age, gender, computer literacy, type of diagnosis and date, was gathered through a survey assessed at the start of the 8 weeks [17,18]. Participants were granted access to the website after providing informed consent and completing baseline data collection. They were informed that they could use the website freely over an 8-week period, choosing how and when to engage with it according to their preferences [17].

To capture the usage by the participants during the 8-week study, there was a continuous collection of log data. Usage refers to how people with dementia and family caregivers engage with the advance care planning website. The log data captured what pages were visited, time spent on each page, interaction with content or functions, frequency of visits to each page, and the search queries used. Upon accessing the website, participants were prompted to identify themselves through a pop-up question, requiring them to fill in their study ID and specify whether they were a person with dementia, a family caregiver, or engaging together as a dyad. A detailed study protocol of the evaluation study is published elsewhere [8].

### Data Analysis

#### Overview

Log data of the advance care planning website were analyzed using the programming language R (version 4.2.3, R Studio). The log data was saved in 3 files, application and access logs and a file to save the interactions with the interactive communication tools. First, the 3 log datasets were cleaned by eliminating irrelevant data, such as admin and php requests. The application logs were further filtered to include only valid study IDs used by the participants. Study IDs were then added to the access logs based on cross matching with IP addresses. The access and application logs were combined, and additional information from the third dataset regarding the completion of interactive communication tools was incorporated by matching IP addresses to study IDs.

#### Assessing the Extent to Which the Website Was Used

First, the interactions with the website were summarized by time spent on the website, pages visited, and who visited. This analysis focused on usage patterns, which provided an initial understanding of the variability in engagement.

#### Identifying Distinct Behavioral Clusters

After, the data underwent K-means clustering. Clustering the data aimed to identify behavioral or interaction patterns that typify user engagement. The 6 features derived from the summary data were used for clustering: total interactions, unique days, duration of use, total clicks on communication, information, and documentation pages. The R package “caret” was used to normalize the data using min-max normalization, converting variables to a range between 0 and 1 [22]. Usage frequency emerged as the key differentiating factor among participants. The “NbClust” package was then used to compare summary statistics and determine the optimal number of clusters, using 30 indices to determine the optimal number of clusters (k) between 2 and 10 based on these metrics [23]. The “NbClust” package indicated that the data could be best classified into 3 clusters, which were named after usage frequency of participants, for example , low, moderate, and high engagement levels. Subsequently, the k-means algorithm was applied to the data for k clusters. Principal component analysis (PCA) was used for data visualization to reduce the multiple features to 2 dimensions. Finally, Kruskal-Wallis rank sum tests and *χ*² tests were conducted to evaluate the significant differences between features for each cluster, providing further insights into the characteristics of the identified user clusters.

### Identification of User Pathways

To visualize how users navigated through the website, we applied process mining techniques using the R package BupaR. This allowed us to map and analyze the various paths users followed across the site’s different sections [24]. Log data were filtered to remove redundant information (taken out: change font size, contrast, privacy policy, read speaker, print, and other). First, a process matrix, which is a 2-dimensional matrix showing the flows between activities, was generated to visualize the entire log dataset [25]. After, individual process maps were produced per study ID to show the path taken by participants. Finally, the individual paths were compared with the IDs per cluster to find similarities and differences between the participants’ paths in each cluster.

### Ethical Considerations

The research protocol was submitted to the Medical Ethics Committee of Brussels university hospital (UZ Brussel) and received ethical approval (BUN 1432022000179). All participants provided informed consent, and the study was conducted in accordance with the relevant ethical guidelines and regulations. To ensure privacy and confidentiality, all participant data were de-identified prior to analysis. Protective measures, such as secure data storage and restricted access to personal information, were implemented to safeguard participant privacy throughout the study.

## Results

### Overview

In total, 52 participants with 31 study IDs were included (21 dyads and 10 family caregivers), consisting of 21 people with dementia and 31 family caregivers. Ten family caregivers participated alone, either because the person with dementia was unable to provide informed consent (6/10) or chose not to participate (4/10). The average age of people with dementia was 62.8 (SD 10.4) years, with 42.8% (9/21) being female. Among family caregivers, 68% (21/31) were female, with an average age of 62.1 (SD 10.9) years. Family caregivers reported an average computer literacy score of 7.5 (on a scale from 1 to 10, with higher scores indicating greater self-reported computer literacy), while people with dementia had an average score of 4 (Table 1).

**Table 1.** Description of the study population.

Variables		Persons with dementia (n=21)	Family caregivers (n=31)
Sex (female), n		9	21
Age (years), mean (SD)		62.1 (10.9)	62.8 (10.4)
Relationship, n			
	Partners^a^	18	25
	Parent (in law)-child	3	6
Profession, n			
	Employed	1	16
	Retired	20^b^	15
Computer literacy^c^, mean (SD)		62.1 (10.9)	62.8 (10.4)
Dementia diagnosis^d^, n			
	Alzheimer disease	15	20
	Vascular dementia	1	3
	Frontotemporal dementia	3	3
	Lewy body dementia	1	1
	Parkinson dementia	0	1
	I do not know	1	3

a Married, living together or in a romantic relationship.

b Six of the retired persons with dementia were forced to take early retirement because of their diagnoses.

c Self-evaluation on a scale from 1 (no computer skills) to 10 (excellent computer skills).

d Dates of diagnoses: January 2013 to December 2022.

### Website Usage

Each study ID (n=31) was logged at least once in the log data, indicating at least 1 visit by 1 or both dyad members and by each family caregiver who participated alone. In 10 study IDs, both the family caregiver and dyad used the website. In 15 instances, only the caregiver accessed it and in 3 cases, solely the dyad engaged with the website. In addition, 3 occurrences involved mixed usage, with indications of the family caregiver, dyad, or person with dementia using the website. The total number of unique interactions by all users (n=31) with the website was 1799 (ie, the total interaction data points), encompassing information searches, clicks, and use of interactive elements. On average, users had 58 (SD 57) interactions over the 8 weeks. The total duration spent on the website during the 8 weeks was 35.3 (SD 82.9) minutes, and, on average, people used the website on 5.5 (SD 3.4) unique days of the 8-week study period.

User interactions revealed that family caregivers had the highest overall number of interactions (757), followed by dyads (235) and people with dementia alone (103). We faced difficulty attributing the information to specific user types for the other 701 interactions documented in the access log file. These unidentified usages per user type arose from difficulties linking interactions with corresponding user types when users left their browsers open for extended periods. The webpage “Advance Care Planning: What Is It?” was visited most (n=304). Followed by the glossary section (209), “Advance Care Planning: Thinking and Talking About Later” (277), “Advance Care Planning: Writing It Down for Later” (259) and the frequently asked questions section (122). The 2 interactive communication tools were used 136 (Thinking Now About Later) and 91 (Levenswensen cards) times.

### Identifying Typical User Behaviors by Clustering

**Figure 1.**
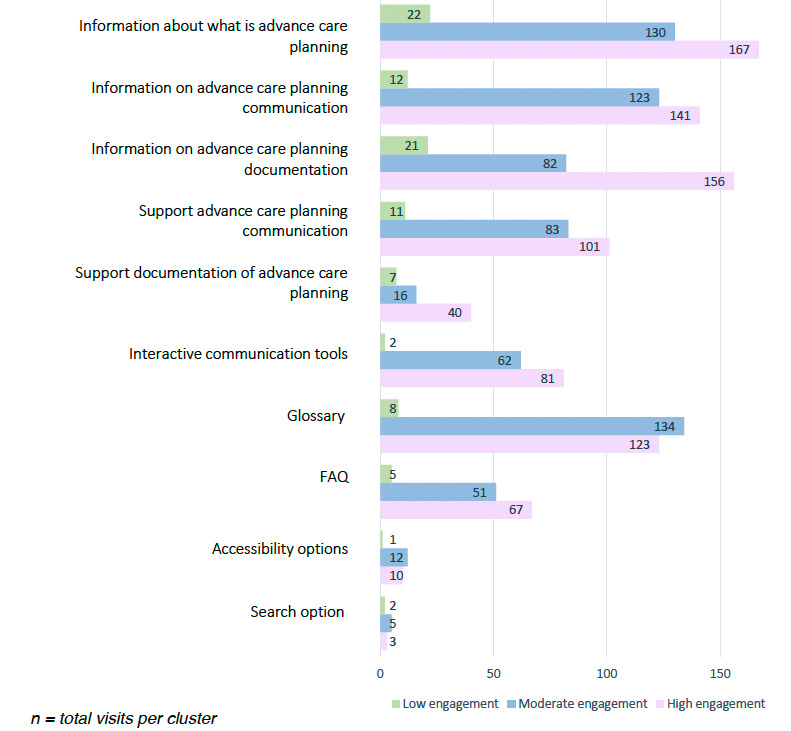
Overview of the most-visited web pages by the 3 clusters. FAQ: frequently asked questions.

To determine the optimal number of clusters, we applied NbClust with 30 indices, revealing that 8 favored 2 clusters and 8 indicated 3 clusters. Following the majority rule, we opted for 3 clusters, each assigned to study IDs reflecting diverse user engagement patterns. Table 2 summarizes each cluster’s characteristics. To showcase the most-visited web pages by the clusters, we categorized all pages into 10 categories (Figure 1; Multimedia Appendix 1 visually shows the web pages corresponding to the categories).

**Table 2.** Overview of the characteristics of use of the website of each cluster.

Characteristics		Moderate engagement level	Low engagement level	High engagement level	*P* value
Size, study ID/n^a^ (%)		15/21 (45)	5/10 (21)	11/16 (34)	—^b^
Total interactions^c^, mean (SD)		50 (13)	21 (9)	86 (15)	<0.001^d^
Unique days, mean (SD)		4.4 (1.5)	1.6 (0.5)	8.7 (3.3)	0.004^d^
Range of use (days), mean (SD)^e^		56.7 (19.3)	9.8 (19.2)	65.6 (16.9)	<0.001^d^
Communication pages visited, mean (SD)		17 (7)	5 (3)	28 (10)	<0.001^d^
Documentation pages visited, mean (SD)		7 (4)	6 (4)	18 (4)	<0.001^d^
Information pages visited, mean (SD)		22 (10)	7 (5)	34 (13)	<0.001^d^
Total number of interactions per user type, n					<0.001^g^
	Caregiver	420	27	310	
	Person with dementia	0	0	103
	Dyad	124	33	88
	Family caregiver and person with dementia^f^	204	54	452

a n is the number of users (N=46 because 5 people with dementia did not use the website or participate anymore).

b Not applicable.

c Interaction is any movement of the user on the website. This can be clicking on a hyperlink, watching a video, opening a page, or printing the web page.

d *χ*² test.

e From the first day of use until the last day. For example, cluster moderate engagement started using the website, and, on average, the last usage was 9.8 days later.

f When the IP address could not be matched with the date or time of the filled-in study ID.

g Kruskal-Wallis rank sum test.

Cluster low engagement (5 participants) exhibited the fewest interactions (mean of 21), shorter duration (mean of 9.8 days), and accessed fewer pages. Cluster moderate engagement (15 participants) had a mean of 50 interactions, visited for 4.4 unique days, with a use duration of approximately 56.7 days (Table 2). Cluster high engagement (11 participants) demonstrated the highest engagement, with the highest total interactions (mean of 86), longer duration (mean of 65.6 days), accessing more diverse pages and including the 3 people with dementia who used the website alone. Statistically significant *P* values underscore distinct engagement patterns among the clusters (Table 2).

Participants across all 3 clusters predominantly visited the “what is advance care planning” web page the most. Low-engagement participants mostly focused on the information pages, especially the documentation information and showed limited interest in interactive tools. Moderate engagement participants visited mostly the advance care planning information pages, frequently using the glossary and accessibility features like contrast and text-to-speech, distinguishing their usage from other clusters. In the high-engagement cluster, participants explored information, communication and documentation pages, actively using interactive communication tools.

### Identification of User Pathways of the Identified User Behavior

**Figure 2.**
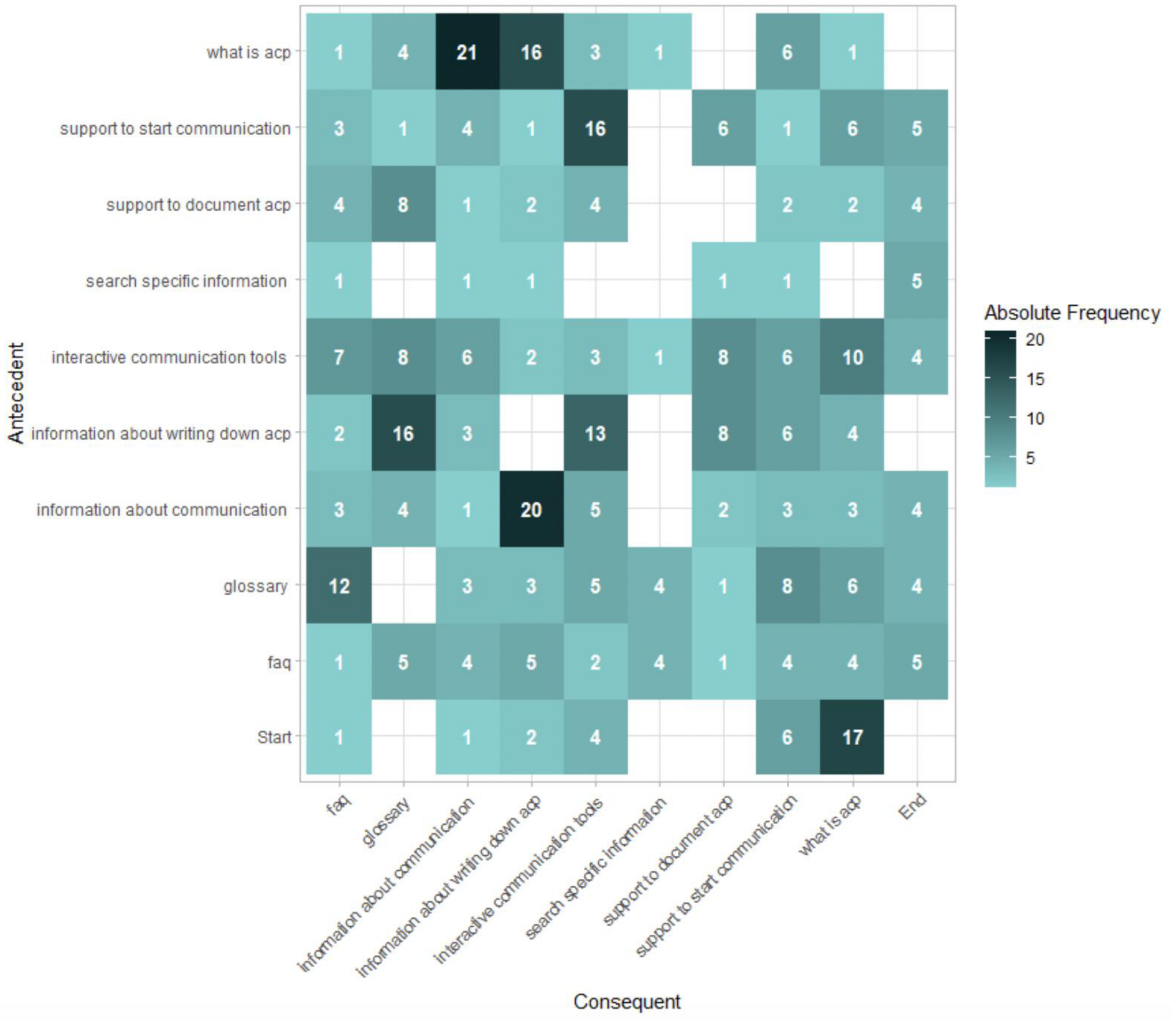
Identification of overall user pathways. acp: advance care planning; FAQ: frequently asked questions.

To identify the user pathways, we first look at the overall pathways of users using a process matrix (Figure 2). The process matrix is a two-dimensional representation that illustrates the flow between the web pages that users have visited. The matrix is organized with antecedent events followed by the consequent events. The primary pathway, observed in 21 instances, initiates with a visit to the “What is advance care planning” page, followed by navigating to a subsequent page providing “information about communication.” Significantly, users frequently started their pathway on informative pages, such as “what is advance care planning,” “information on communication,” or “Information on documentation.” Subsequently, they progress to explore additional information pages. Noteworthy is the observation that users typically visit pages providing information about communication or documentation before engaging with the interactive communication tools.

**Figure 3.**
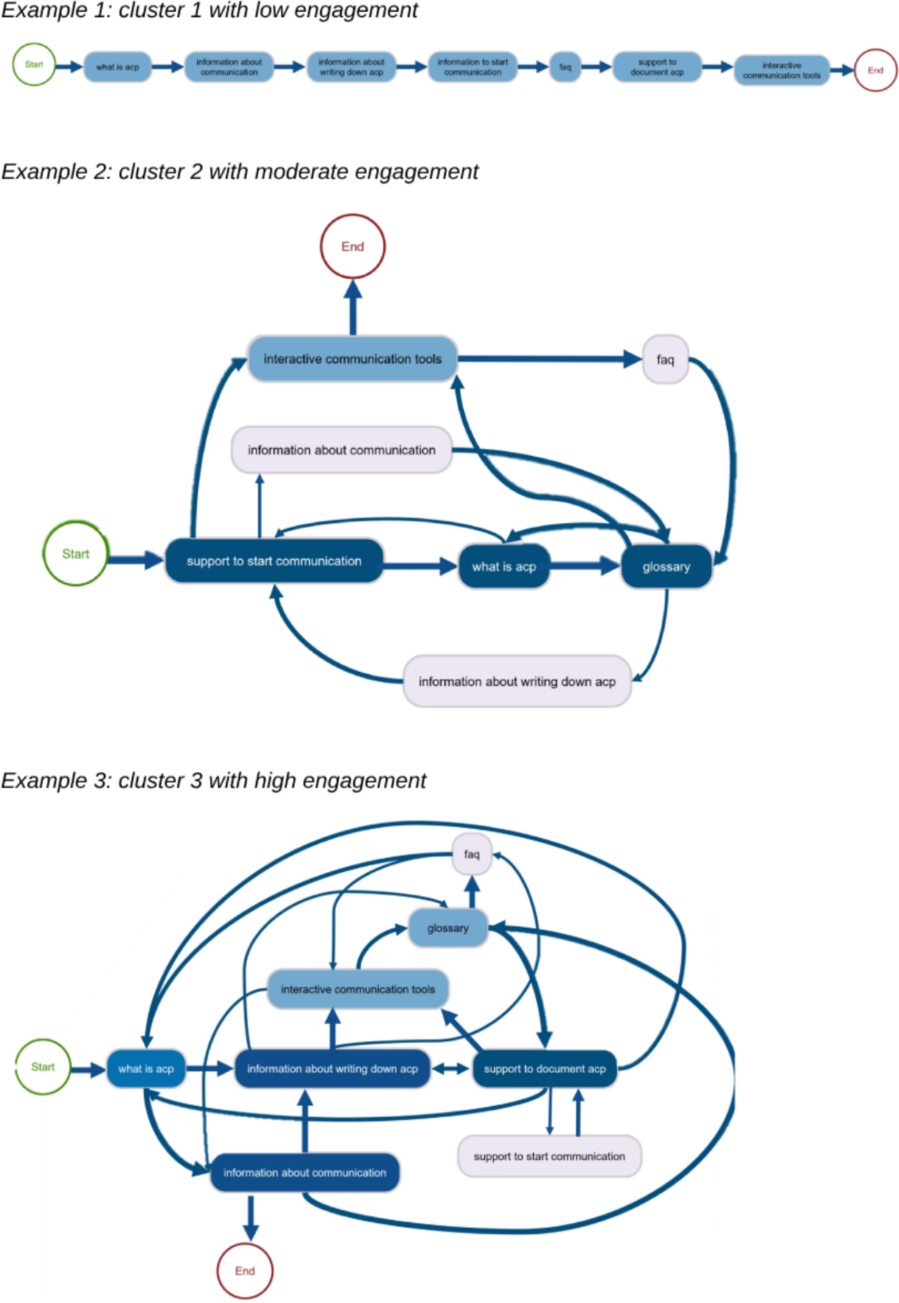
Examples of the 3 user paths. acp: advance careplanning; faq: frequently asked questions.

Then, the user pathways of the identified user behavior clusters were identified (Figure 3). Low-engagement participants (Figure 3, example 1) displayed a linear and direct browsing style, rarely revisiting previous pages during navigation. In contrast, the participants with moderate and high engagement (Figure 3, examples 2 and 3) explored the website by visiting pages sequentially, occasionally revisiting previously viewed pages. High engagement extensively explored various pages, moving between information and guidance pages. Participants with moderate engagement involved frequent transitions between pages but less often than those with high engagement.

## Discussion

### Principal Findings

The analyses of the log data of an 8-week evaluation study of a website to support people with dementia and their families in advance care planning showed that family caregivers used the website most often, either alone or with a person with dementia, that is, dyad. Three distinct engagement patterns emerged in this study: low, moderate, and high. Low-engagement users tended to follow a more linear path on the website, while moderate- and high-engagement users displayed a more dynamic engagement, exploring the website in diverse ways.

Flexible user navigation patterns were evident in our study, challenging the conventional linear advance care planning representations found in other web-based tools in which users go through a stepwise process, typically starting with information provision, prompting reflection, moving on to communication, and concluding with documentation in the form of advance directives [15]. This rigid structure, embedded in the design of other ACP websites, may restrict easy access to subsequent features or content until earlier steps are completed. While this structured pathway might work for some users, it assumes that everyone is ready to follow the same route through the ACP process, which may not always be the case [7,8,26]. In our study, low-engagement users tended to follow a more linear pathway. However, those with moderate or high engagement demonstrated more dynamic and varied usage patterns. Some users initiated their engagement by seeking information, while others prioritized documentation or directly accessed communication tools. This flexibility of navigation aligns with feedback from family caregivers during the website’s development process, which emphasized the importance of allowing users to navigate at their own pace, rather than following a rigid, linear path [8]. This flexibility allowed for personalized engagement, as different users may be at different stages of readiness to engage with sensitive topics, such as future care planning. Furthermore, this approach aligns with broader technological research [7,10,26], which shows that family caregivers prefer flexible navigation in internet-based tools [27,28]. These findings suggest a need for a more flexible approach, indicating that users should have the freedom to navigate tools that align best with their needs, which is also supported by other research [5,26,29]. This also aligns with Belgian clinical guidelines [30], explicitly mentioning that health care professionals should tailor advance care planning in dementia, including style and content, to the “person’s level and rhythm.”

Despite participants’ expressed interest in advance care planning in the evaluation study, people with dementia rarely engaged with the website on their own. Family caregivers and the person with dementia did engage together, emphasizing the family’s importance. This finding is not necessarily surprising as much literature points at the importance of family in a dementia trajectory. A recently published consensus definition on advance care planning in dementia also highlighted family as highly important and specific in this population [6,31]. Regarding the use or uptake of websites among people with dementia, involving family caregivers can play a facilitating role; however, it is essential to acknowledge that not all people with dementia have family caregivers or families directly engaged in their care [32]. Furthermore, overreliance on family caregivers may unintentionally hinder independent usage, potentially undermining autonomy. Therefore, achieving an inclusive environment necessitates balancing involving family caregivers and promoting self-usage.

### Strengths and Limitations of the Study

This study has several strengths that contribute to the robustness of our study’s findings. Using log data, this study is the first to examine user engagement of people with dementia and their families with a website to support advance care planning. It offers a comprehensive understanding of their specific usage patterns. The elimination of recall bias is another key strength, as log data provided an accurate account of how users engaged with the website. The study also has limitations with regard to the data used. We encountered difficulty identifying the specific type of user for all log data because not all access logs could be matched with the date or time of the filled-in type of user in the application log. In addition, due to a 1-month retention period for log data, a small portion of data (7 days with interactions from 2 users, as indicated in Google Analytics) was lost as it was not downloaded before deletion from the server. Another limitation is the relatively small dataset for our log data analyses. Specifically, the number of participants with dementia who accessed the website independently was small what limits our ability to make comparisons in usage patterns between people with dementia, family caregivers, and dyads. Further research with a larger sample of people with dementia, including both independent and caregiver-supported users, is needed to enhance the robustness of the findings and provide stronger insights into their engagement patterns. Finally, a limitation arises from self-selection bias in user type; for example, users might identify as “family caregivers” while engaging together, and vice versa, introducing variability that could impact the accuracy of findings.

### Conclusions

This study offers insights in how people with dementia and their family caregivers use a website designed to support them in advance care planning. The findings show that family caregivers are the website’s primary users, often engaging with the website alone or together with the person with dementia. Three distinct user engagement patterns, low, moderate, and high engagement, were identified, with more dynamic navigation observed among high-engagement users, particularly those using interactive communication tools. These findings underscore the need for flexible user pathways in online advance care planning tools, allowing personalized navigation. Future research with a larger and more diverse sample of persons with dementia is necessary to confirm these findings and allow for more detailed comparisons across different user groups.

## Supplementary material

10.2196/60652 Multimedia Appendix 1 Overview of the website.

10.2196/60652 Multimedia Appendix 2 The interactive communication tools.

## Abbreviations

ACP: advance care planning
