# Supplementary material for: Uncovering Specific Navigation Patterns by Assessing User Engagement of People With Dementia and Family Caregivers With an Advance Care Planning Website: Quantitative Analysis of Web Log Data
Source: JMIR Aging. 2025 Feb 11;8:e60652. doi: 10.2196/60652 (PMC11835784; doi:10.2196/60652)

**Appendix 1 – overview of the website**

*Note that the original language is Dutch. The English version is translated by the automatic translate function in Google Chrome.*

The home page:


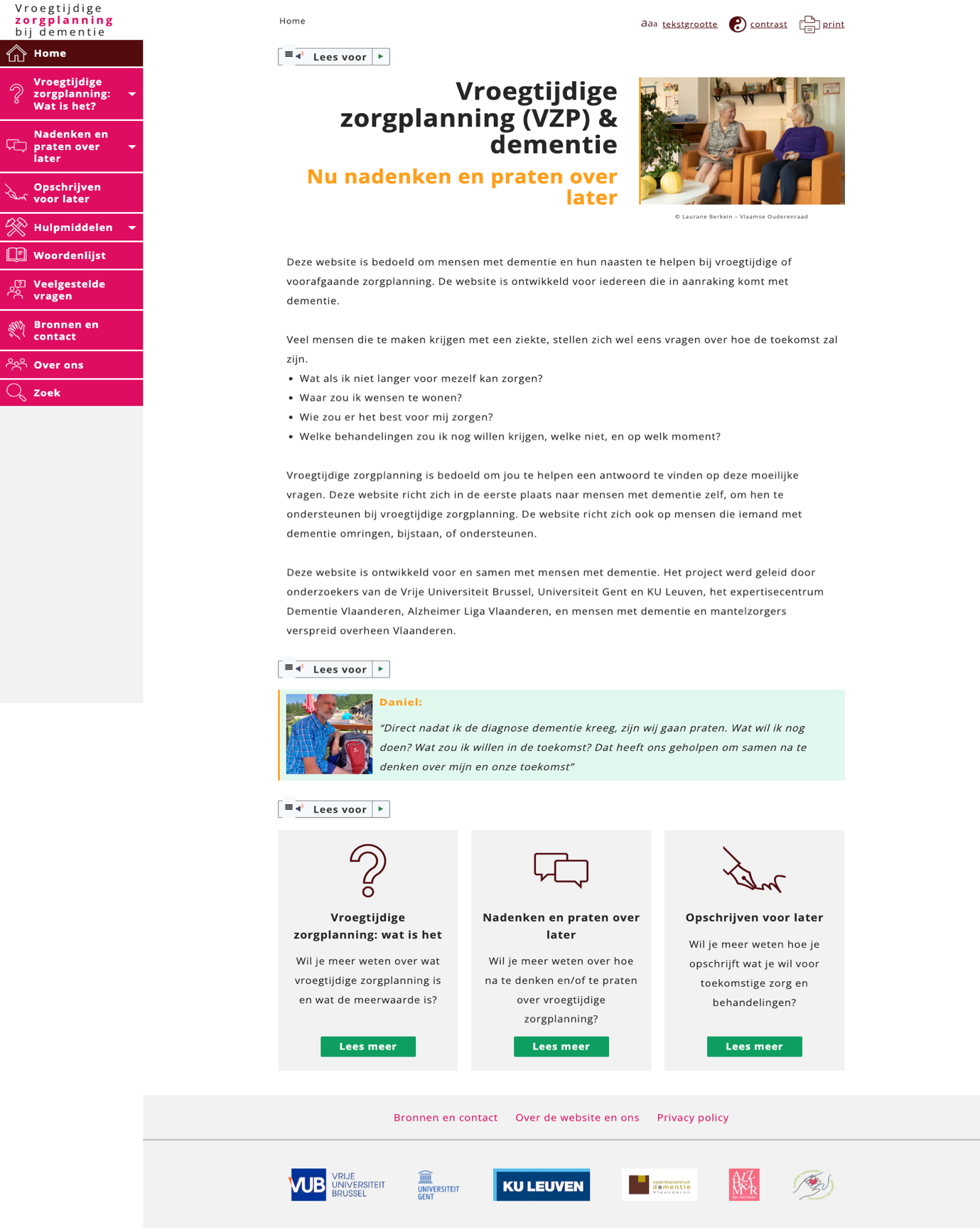


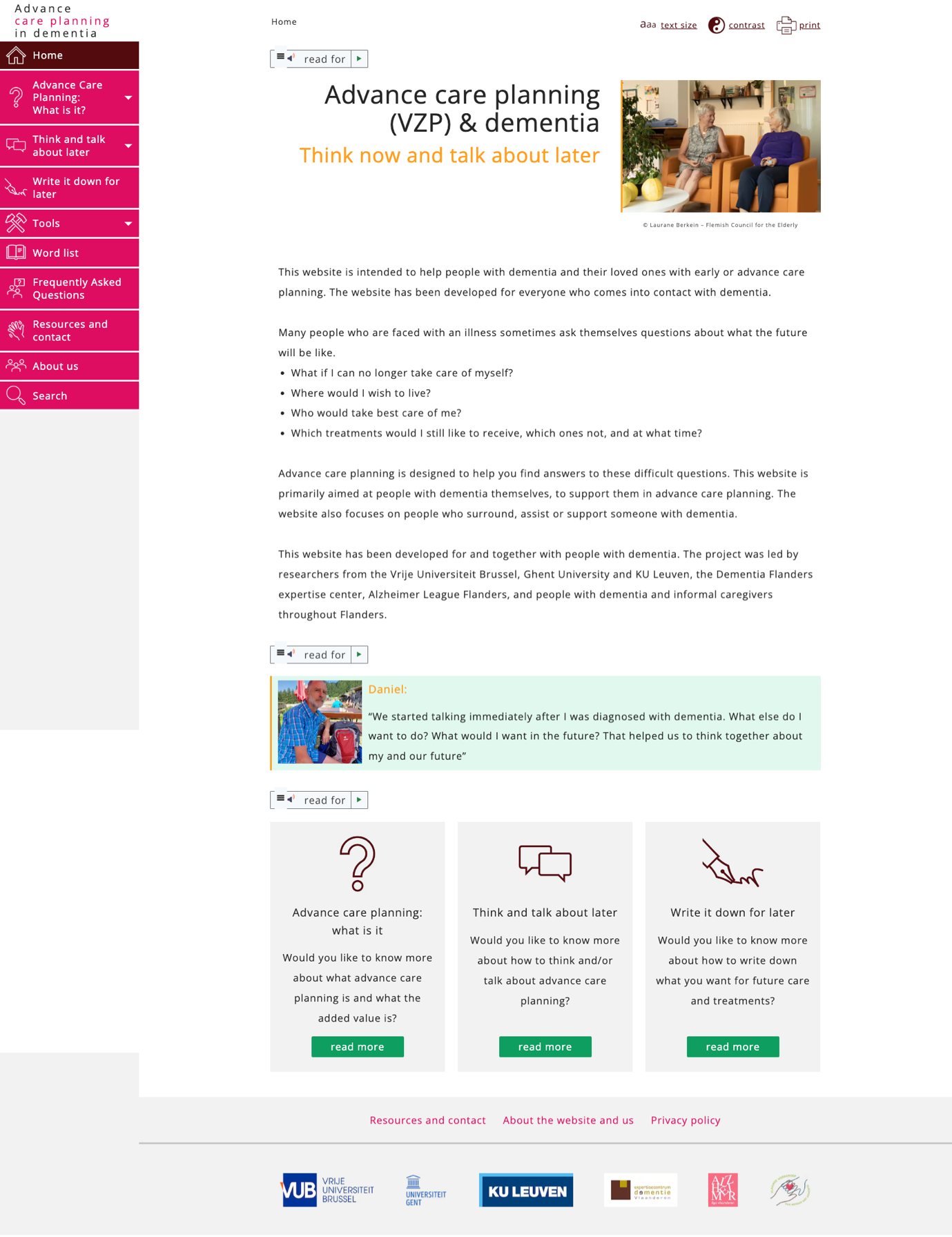


Example page: thinking and talking about advance care planning


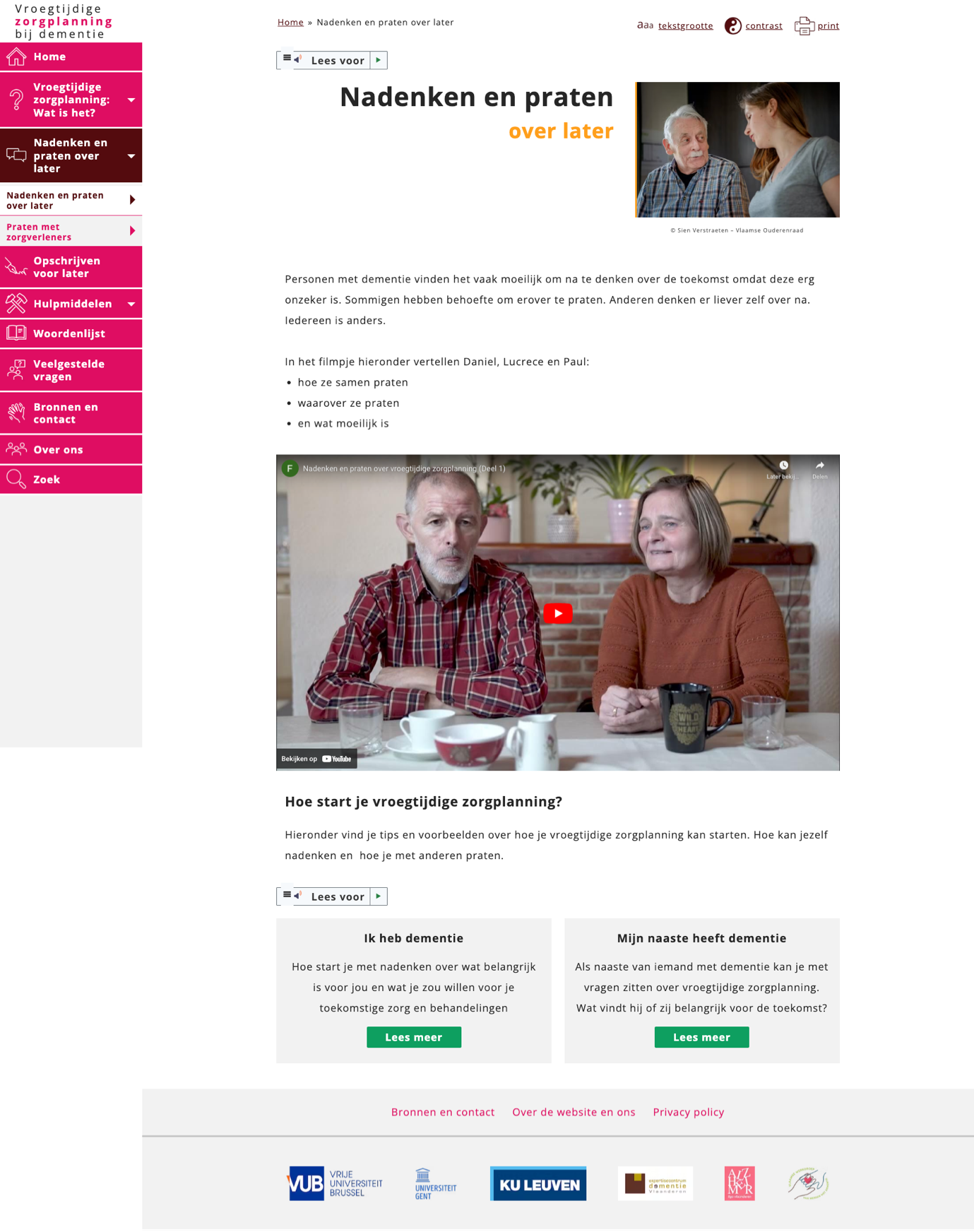


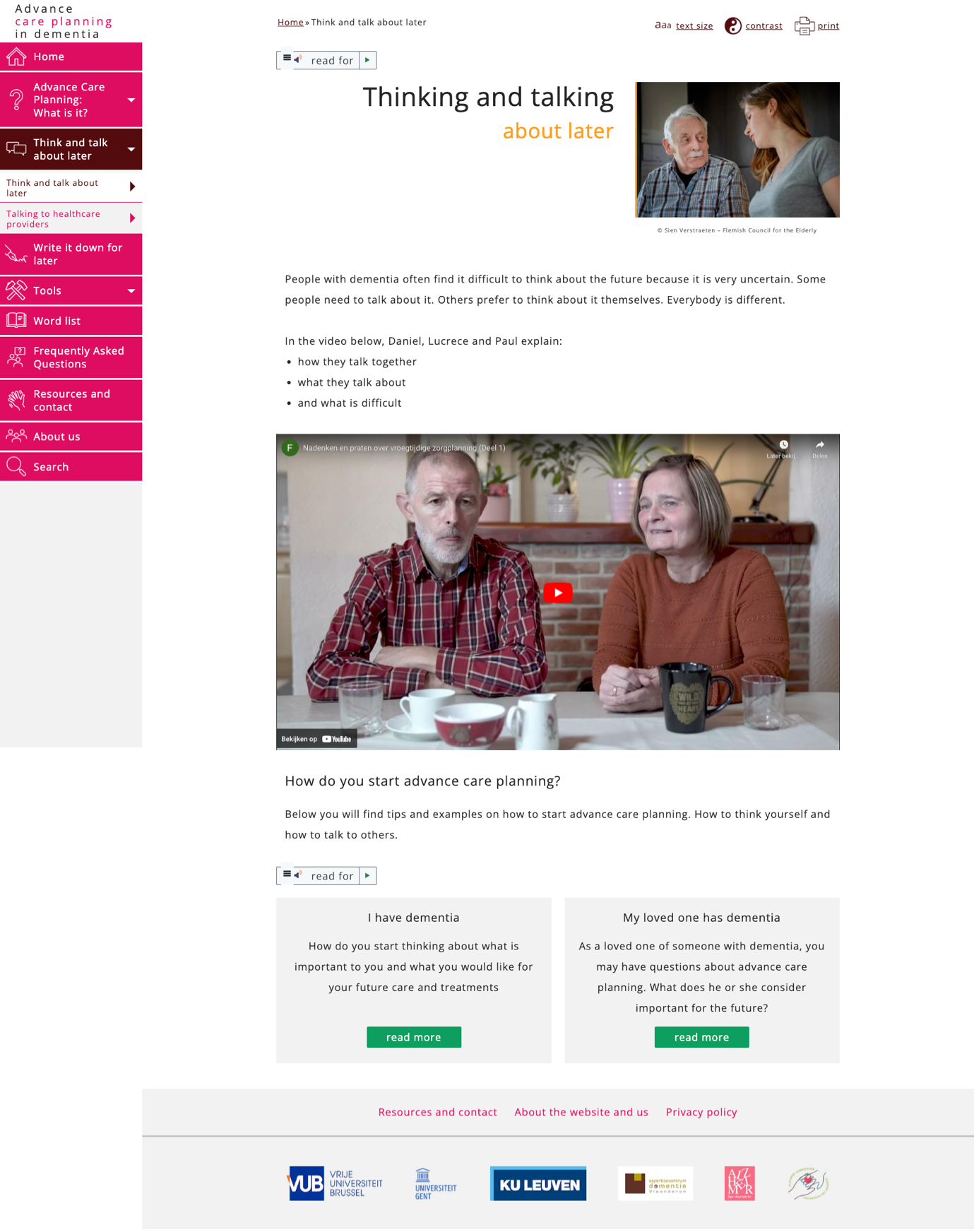


Example page: frequently asked questions


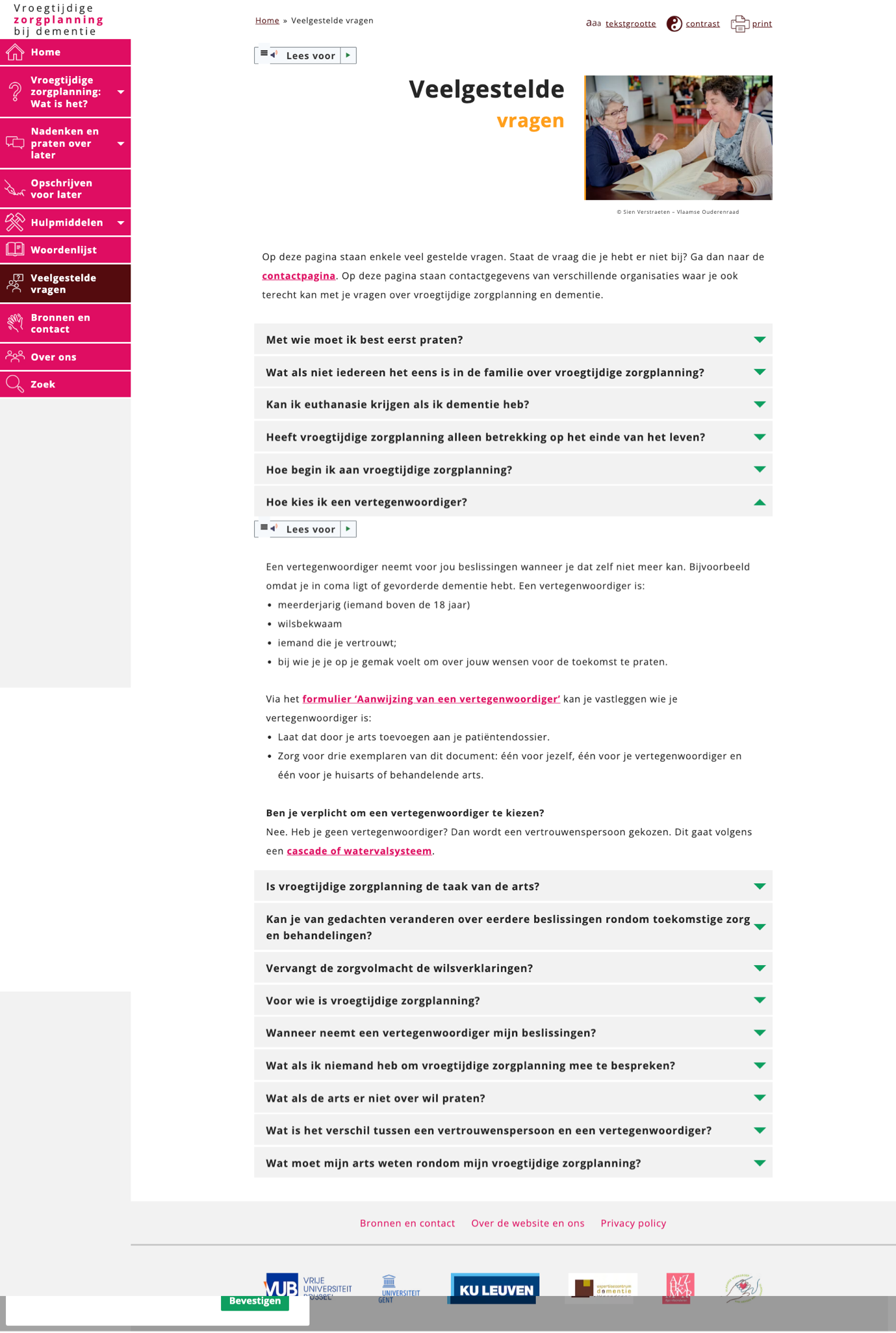


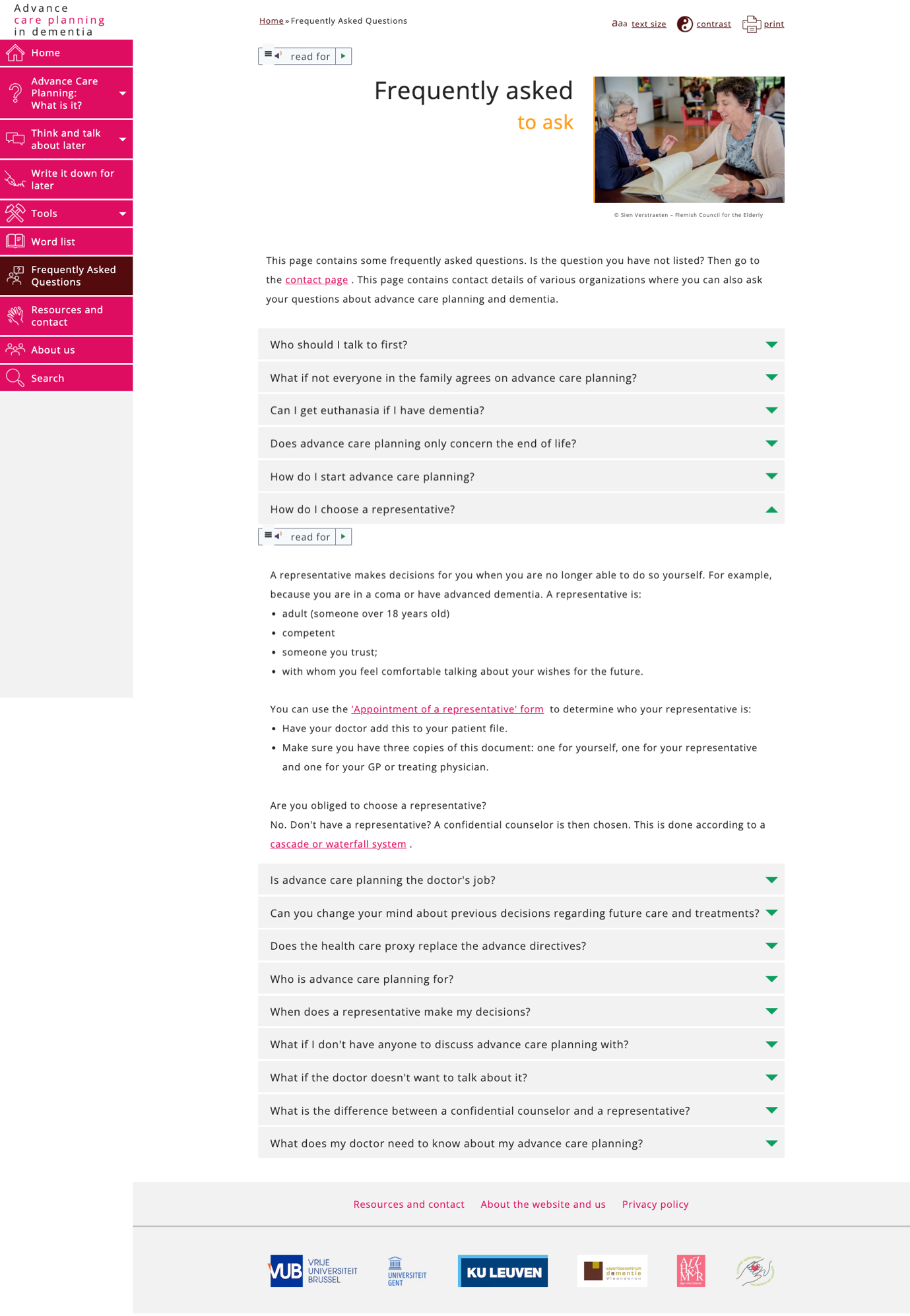

Supplement: Multimedia Appendix 1 [file aging-v8-e60652-s001.docx]
