# Supplementary material for: Uncovering Specific Navigation Patterns by Assessing User Engagement of People With Dementia and Family Caregivers With an Advance Care Planning Website: Quantitative Analysis of Web Log Data
Source: JMIR Aging. 2025 Feb 11;8:e60652. doi: 10.2196/60652 (PMC11835784; doi:10.2196/60652)

**Appendix 2 – the interactive communication tools**

*Preview of the Interactive Card Tool “Life Wishes”.*


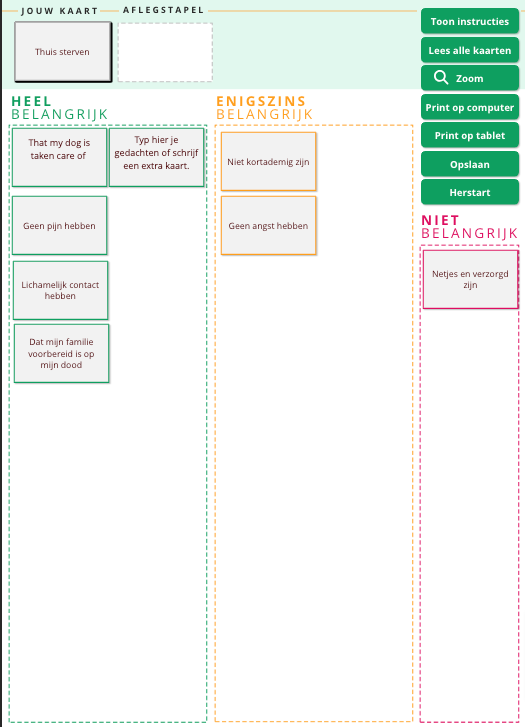


*Example of the Interactive Writing Tool “Thinking Now About Later”*


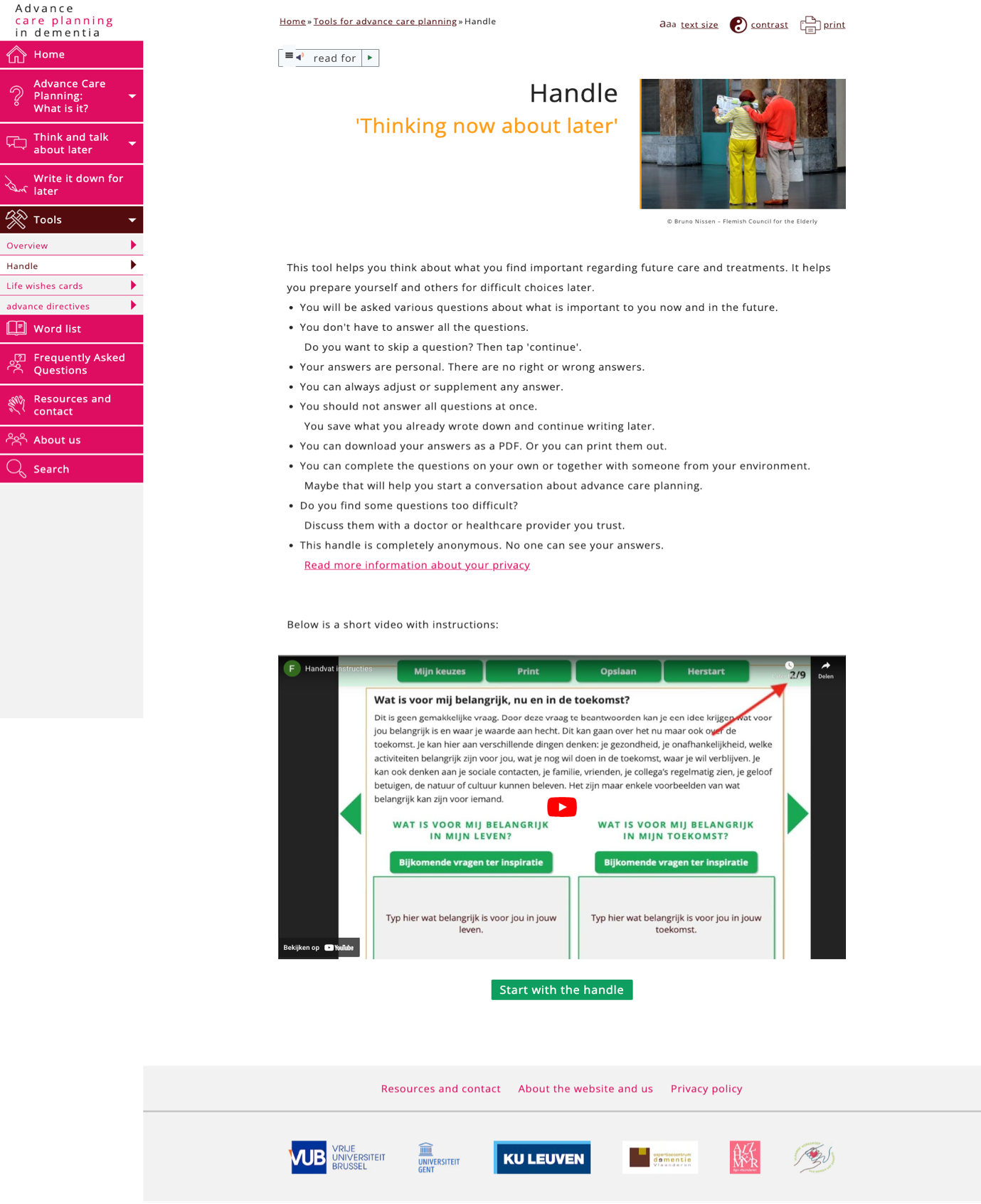


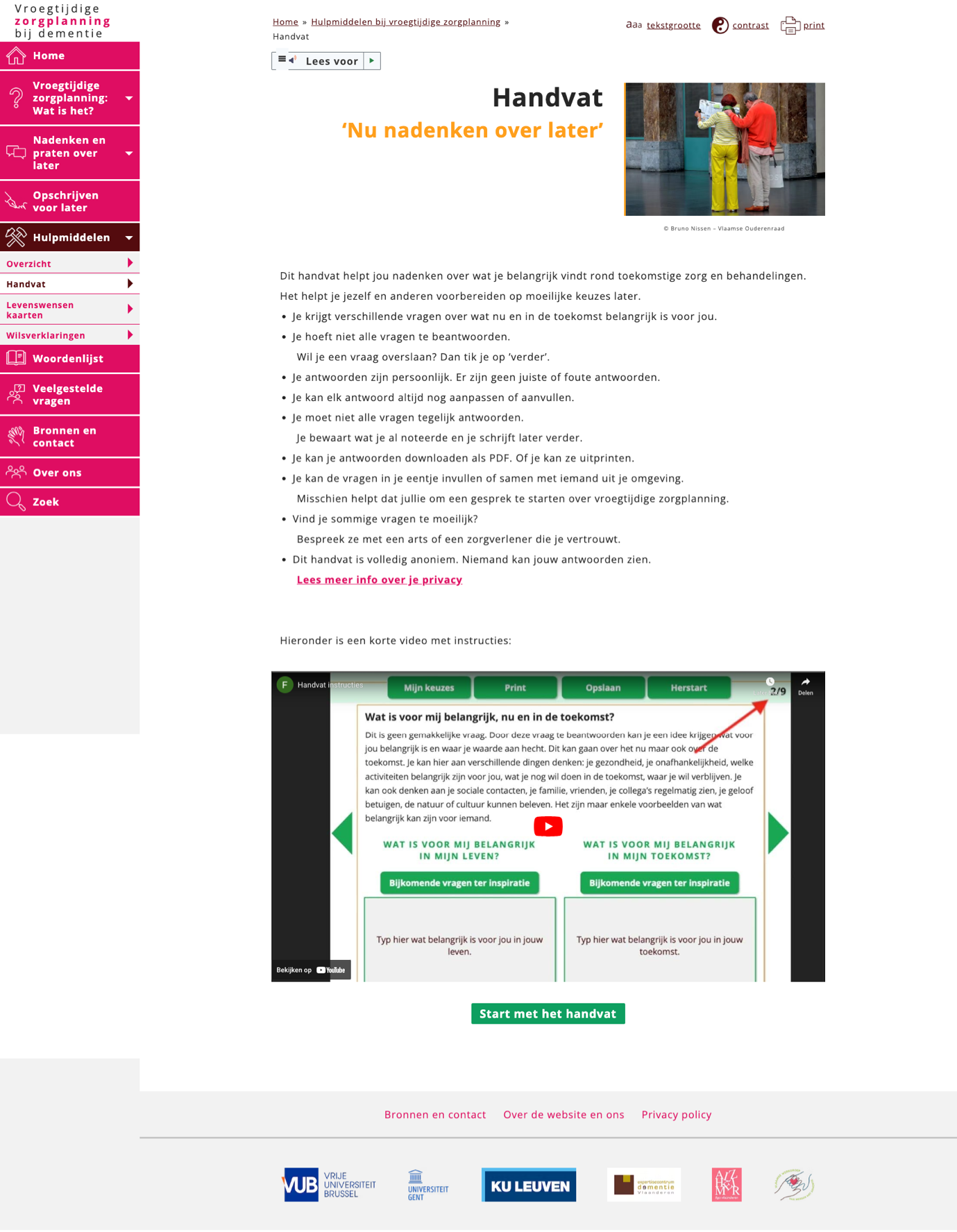


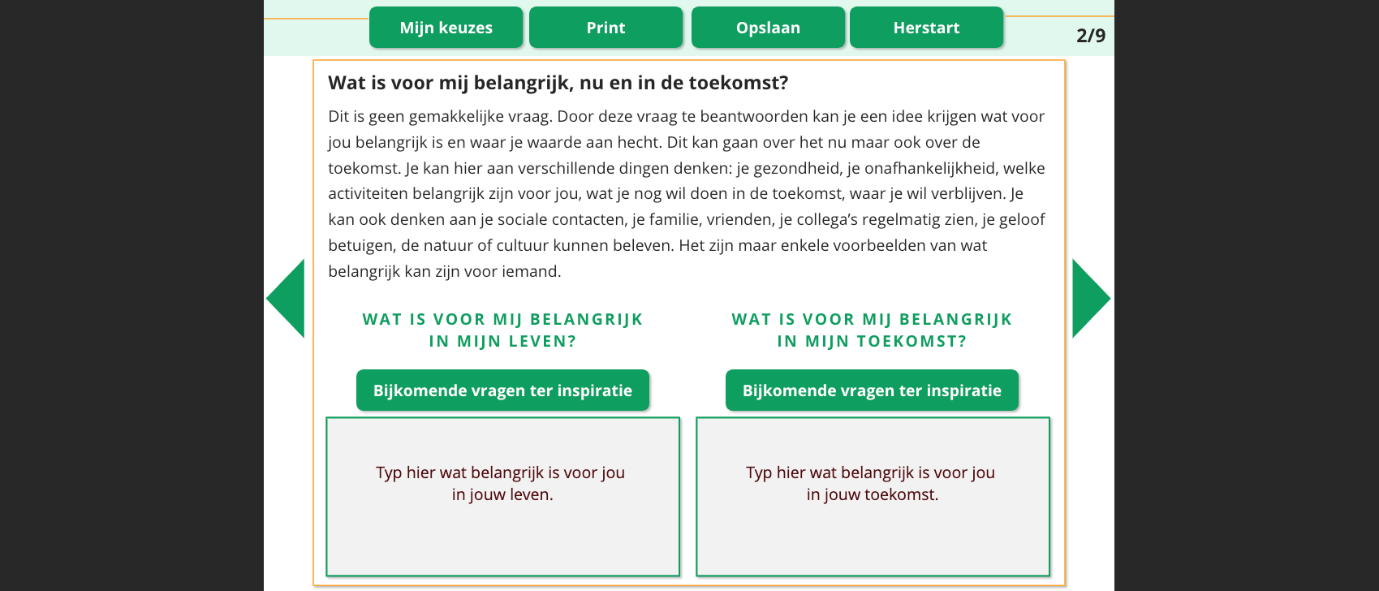

Supplement: Multimedia Appendix 2 [file aging-v8-e60652-s002.docx]
